# The Prognostic Value of the Advanced Lung Cancer Inflammation Index on Disease-Free Survival in Patients with Gastric Cancer Receiving Neoadjuvant Chemotherapy

**DOI:** 10.3390/jcm15176806

**Published:** 2026-09-02

**Authors:** Mahmut Kara, Muslih Ürün, Senar Ebinç, Yasin Sezgin, Yonca Yılmaz Ürün, Mehmet Naci Aldemir

**Affiliations:** Department of Medical Oncology, Faculty of Medicine, Van Yuzuncu Yil University, Van 65090, Türkiye

**Keywords:** gastric cancer, Advanced Lung Cancer Inflammation Index (ALI), neoadjuvant chemotherapy, disease-free survival

## Abstract

**Objectives:** The present study investigated whether the pre-treatment Advanced Lung Cancer Inflammation Index (ALI) could serve as a prognostic indicator of disease-free survival (DFS) in patients with locally advanced gastric cancer treated with neoadjuvant chemotherapy followed by curative D2 gastrectomy. **Methods:** We retrospectively analyzed 157 patients with gastric adenocarcinoma who received treatment between 2016 and 2025. The pre-treatment ALI was derived from body mass index, serum albumin concentration, and the neutrophil-to-lymphocyte ratio. The association of clinical and laboratory variables with DFS was assessed using Kaplan–Meier survival analysis and Cox proportional hazards regression. **Results:** Median patient age was 63.6 years (61.1% male). Overall, 50.3% of patients experienced recurrence. Multivariate analysis demonstrated that ALI is an independent prognostic factor for DFS (HR = 0.867; 95% CI: 0.768–0.978; *p* = 0.020). Advanced age (*p* = 0.040), advanced clinical N stage (*p* < 0.001), and non-FLOT regimens (*p* = 0.003) also significantly predicted poorer DFS. Kaplan–Meier analysis confirmed that median DFS was significantly shorter in the low ALI group (38.9 months) compared to the high ALI group (52.0 months; *p* = 0.029). **Conclusions:** A low pre-treatment ALI score independently predicts shorter DFS in locally advanced gastric cancer patients treated with neoadjuvant chemotherapy and surgery. ALI serves as an accessible, low-cost biomarker to identify high-risk patients, warranting further prospective validation.

## 1. Introduction

Despite substantial improvements in the diagnosis and management of gastric cancer, it continues to be associated with considerable mortality [1]. While it may present with clinical symptoms such as loss of appetite, weight loss, abdominal pain, and difficulty swallowing, it can also be asymptomatic in the early stages [2]. Currently, neoadjuvant therapy is administered for locally advanced disease. The goal of this treatment is to reduce tumor size, increase the likelihood of R0 resection, and prevent micrometastases [3]. Postoperative chemotherapy has also become standard practice. However, despite completing postoperative treatments, some patients experience recurrence, which negatively affects disease-free survival (DFS) and overall survival. For this reason, studies have been conducted to determine which patients are at high risk for recurrence following neoadjuvant therapy [4].

In clinical practice, the most common factor used to determine prognosis and recurrence is the tumor’s TNM (tumor size-lymph node-metastasis) staging. In particular, the T and N stages of the tumor are among the most important factors determining the course of the disease [5]. While factors such as age and the Lauren histological subtype of the tumor are also noted to be influential, studies indicate that tumor markers such as Carbohydrate Antigen 19-9 (CA19-9) may also play a role in prognosis and recurrence [6,7]. However, these parameters are also insufficient for predicting the course of the disease. Studies conducted in recent years have examined the relationship between cancer prognosis and malnutrition and systemic inflammation. In this context, there are studies on the prognostic value of inflammation [8]. The Advanced Lung Cancer Inflammation Index (ALI), initially developed and investigated in patients with non-small cell lung cancer (NSCLC), is a composite biomarker that reflects both nutritional status and systemic inflammation [9]. The success of this biomarker in NSCLC has paved the way for its investigation in other cancers as well [10,11]. Previous studies have investigated the prognostic significance of the ALI score in patients with gastric cancer undergoing surgical resection and subsequent adjuvant treatment [12,13,14]. In contrast, evidence regarding its ability to predict disease recurrence among patients treated with neoadjuvant therapy followed by surgery remains scarce.

This study aimed to evaluate the association between pretreatment ALI and disease-free survival in gastric cancer patients treated with neoadjuvant chemotherapy followed by curative gastrectomy and D2 lymph node dissection. Additionally, the study sought to investigate the prognostic impact of other clinical-pathological and treatment-related factors on disease-free survival (DFS).

## 2. Materials and Methods

### 2.1. Study Design and Patient Population

This retrospective observational study included patients with gastric cancer who underwent neoadjuvant chemotherapy followed by surgical resection. Medical records of patients treated and subsequently followed at the Medical Oncology Clinic of Van Yüzüncü Yıl University Faculty of Medicine Hospital between January 2016 and December 2024 were retrospectively reviewed.

The study included patients aged 18 years and older who had a histopathologically confirmed diagnosis of gastric adenocarcinoma, clinically resectable or locally advanced disease, received neoadjuvant chemotherapy, and underwent surgery and D2 dissection. Patients with distant metastases at the time of diagnosis, those who did not undergo surgery after neoadjuvant chemotherapy, those who underwent D1 dissection, those with incomplete follow-up data, and those lacking the laboratory or anthropometric data required to calculate the ALI score were excluded from the study.

Among patients who received neoadjuvant chemotherapy, 11 did not subsequently undergo surgical resection and were excluded from the study. Five patients remained medically and surgically eligible for resection but declined surgery because of concerns and fear regarding the surgical procedure. Six patients developed disease progression during or following neoadjuvant chemotherapy and consequently became unresectable.

### 2.2. Clinical and Pathological Data

The patients’ demographic, clinical, laboratory, treatment, and pathological characteristics were obtained retrospectively from patient charts and the hospital record system. Age, sex, ECOG performance status, tumor location, histological subtype, tumor grade, clinical T and N stages, neoadjuvant chemotherapy regimen, number of neoadjuvant cycles received, tumor regression score, number of lymph nodes resected, recurrence status, and final status were recorded.

The laboratory variables assessed in this study comprised hemoglobin, total white blood cell count, absolute neutrophil, lymphocyte, monocyte, and platelet counts, serum albumin, carcinoembryonic antigen (CEA), and carbohydrate antigen 19-9 (CA19-9) levels. Ki-67 proliferation index and HER2 status were also documented when available. Body mass index (BMI) was determined as body weight in kilograms divided by height in meters squared.

### 2.3. Calculation of the ALI Score

The Advanced Lung Cancer Inflammation Index (ALI) was determined from the pre-treatment body mass index (BMI), serum albumin concentration, and neutrophil-to-lymphocyte ratio (NLR). The index was calculated according to the following formula:

ALI = (BMI × serum albumin)/NLR

In this calculation, NLR represents the ratio of the absolute neutrophil count to the absolute lymphocyte count.

Alternatively, the formula was expressed as follows:

ALI = BMI × serum albumin × lymphocyte count/neutrophil count

The ALI score was evaluated as a continuous variable in Cox regression analyses. To enhance clinical interpretability, the ALI was analyzed in 10-unit increments. In the Kaplan–Meier analysis, patients were divided into low-ALI and high-ALI groups based on the median ALI value. The median ALI value was determined to be 39.87.

### 2.4. Survival Definitions

The primary endpoint of the study was disease-free survival (DFS). For the purposes of this study, DFS was defined as the interval from the date of initial histopathological diagnosis to the first documented disease recurrence following curative surgery. Patients who did not experience recurrence were censored at the date of their last follow-up.

### 2.5. Statistical Analysis

Statistical analyses were performed using IBM SPSS Statistics version 24.0 (IBM Corp., Armonk, NY, USA). According to their distribution, continuous variables were summarized using medians and ranges, whereas categorical variables were reported as frequencies and percentages. The ability of the ALI score to discriminate between patients with and without disease recurrence was assessed by receiver operating characteristic (ROC) curve analysis.

Cox regression analysis was performed to identify factors affecting disease-free survival (DFS). Univariate Cox regression analyses were conducted first. Covariates demonstrating a *p*-value below 0.10 in the univariate analysis were subsequently entered into the multivariable Cox proportional hazards regression model. ALI was evaluated as a continuous variable in the multivariate model, with each 10-unit increase considered. The results of the Cox regression analyses were expressed as hazard ratios (HRs) with corresponding 95% confidence intervals (CIs) and *p*-values.

As a sensitivity analysis, tumor regression grade (TRG), a post-treatment pathological response variable, was additionally incorporated into the primary multivariable Cox proportional hazards model to assess whether the association between pretreatment ALI and DFS remained robust after adjustment for pathological treatment response. TRG was modeled as a categorical variable, with TRG1 used as the reference category.

Disease-free survival (DFS) was estimated using the Kaplan–Meier method, with survival distributions between the ALI groups compared by the log-rank test. Statistical significance was defined as a two-sided *p*-value below 0.05.

## 3. Results

### 3.1. Patient Characteristics

The study cohort comprised 157 patients, with a median age of 63.55 years (range, 32.65–86.72 years). There were 96 men (61.1%) and 61 women (38.9%). Regarding ECOG performance status, 82 patients (52.2%) had a score of 0, 70 (44.6%) had a score of 1, and 5 (3.2%) had a score of 2. Histologically, non-signet ring cell tumors were identified in 128 patients (81.5%), whereas 29 patients (18.5%) had signet ring cell histology. Tumor differentiation was classified as grade 1 in 8 patients (5.1%), grade 2 in 96 (61.1%), and grade 3 in 53 (33.8%).

Tumor location was at the gastroesophageal junction in 26 patients (16.6%), the fundus in 50 patients (31.8%), the corpus in 53 patients (33.8%), and the antrum in 28 patients (17.8%). In terms of clinical T stage, 8 patients were classified as cT1 (5.1%), 52 as cT2 (33.1%), 67 as cT3 (42.7%), and 30 as cT4 (19.1%). The clinical N stage was cN0 in 21 patients (13.4%), cN1 in 64 patients (40.8%), cN2 in 39 patients (24.8%), and cN3 in 33 patients (21.0%).

As a neoadjuvant treatment regimen, 125 patients received FLOT (79.6%) and 32 patients received a non-FLOT regimen (20.4%). Patients received a median of four cycles of neoadjuvant chemotherapy. According to the tumor regression grading system, 37 patients (23.6%) were classified as TRG1, 61 (38.9%) as TRG2, and 59 (37.6%) as TRG3. The median number of lymph nodes retrieved during surgery was 28.

When laboratory and inflammatory-nutritional parameters were examined, the median CEA level was 3.52, the median CA19-9 level was 12.0, the median albumin level was 3.95 g/dL, and the median BMI was 24.77 kg/m^2^. The median ALI score was calculated as 39.87, and ALI values ranged from 4.79 to 132.58. During the follow-up period, 79 patients experienced a recurrence (50.3%), while 78 patients did not (49.7%). At the time of the final assessment, 93 patients were alive (59.2%), and 64 patients had died (40.8%) (Table 1).

### 3.2. The Relationship Between ALI Score and DFS

In the ROC analysis, the ALI score showed limited discriminatory ability for predicting recurrence. Because lower ALI values were hypothesized to be associated with a higher risk of recurrence, the ROC analysis was interpreted with low ALI as the positive state. Under this direction, the AUC was 0.576, indicating limited discriminatory performance. The ROC analysis was therefore not used to define a prognostic cutoff for ALI. Instead, ALI was evaluated as a continuous variable in Cox regression analyses, and patients were divided according to the median ALI value for the Kaplan–Meier analysis.

In univariate Cox regression analysis, ALI was found to be significantly associated with DFS. Each 10-unit increase in ALI was associated with an approximately 12.1% reduction in the risk of recurrence. In the univariate analysis, the HR for ALI was 0.879, with a 95% CI of 0.796–0.971 and a *p*-value of 0.011.

In the multivariable Cox proportional hazards analysis, adjustment for sex, age, clinical T and N stages, tumor grade, neoadjuvant treatment regimen, and CEA and CA19-9 concentrations showed that ALI was independently associated with DFS. For every 10-unit increase in the ALI score, the hazard of recurrence decreased by approximately 13.3%. The adjusted hazard ratio for ALI was 0.867 (95% CI, 0.768–0.978; *p* = 0.020).

In the multivariate analysis, other factors independently associated with DFS were advanced age, advanced clinical N stage, and a neoadjuvant treatment regimen other than FLOT. The risk of recurrence increased with advancing age (HR 1.027, 95% CI 1.001–1.054; *p* = 0.040). Patients with a high clinical N stage had a higher risk of recurrence (HR 2.709, 95% CI 1.584–4.634; *p* < 0.001). Patients who received neoadjuvant therapy other than FLOT also had a higher risk of recurrence compared to those who received FLOT (HR 2.116, 95% CI 1.293–3.464; *p* = 0.003). Clinical T stage, tumor grade, CEA, and CA19-9 were significant in univariate analysis but lost their independent significance in multivariate analysis (Table 2).

### 3.3. Sensitivity Analysis Including Tumor Regression Grade

To evaluate the robustness of the prognostic association between pretreatment ALI and DFS after accounting for pathological response to neoadjuvant treatment, an additional sensitivity analysis was performed by incorporating TRG into the multivariable Cox proportional hazards model. After additional adjustment for TRG, ALI remained significantly associated with DFS (HR = 0.873, 95% CI: 0.774–0.985; *p* = 0.028 per 10-unit increase). The magnitude and direction of this association were similar to those observed in the primary multivariable model (HR = 0.867, 95% CI: 0.768–0.978; *p* = 0.020). Compared with TRG1, the HRs were 2.088 for TRG2 (95% CI: 0.875–4.984; *p* = 0.097) and 2.165 for TRG3 (95% CI: 0.840–5.580; *p* = 0.110). These findings support the robustness of the association between pretreatment ALI and DFS after additional adjustment for pathological treatment response. The results of the sensitivity analysis are presented in Supplementary Table S1.

### 3.4. Kaplan–Meier Survival Analysis

**Figure 1:**
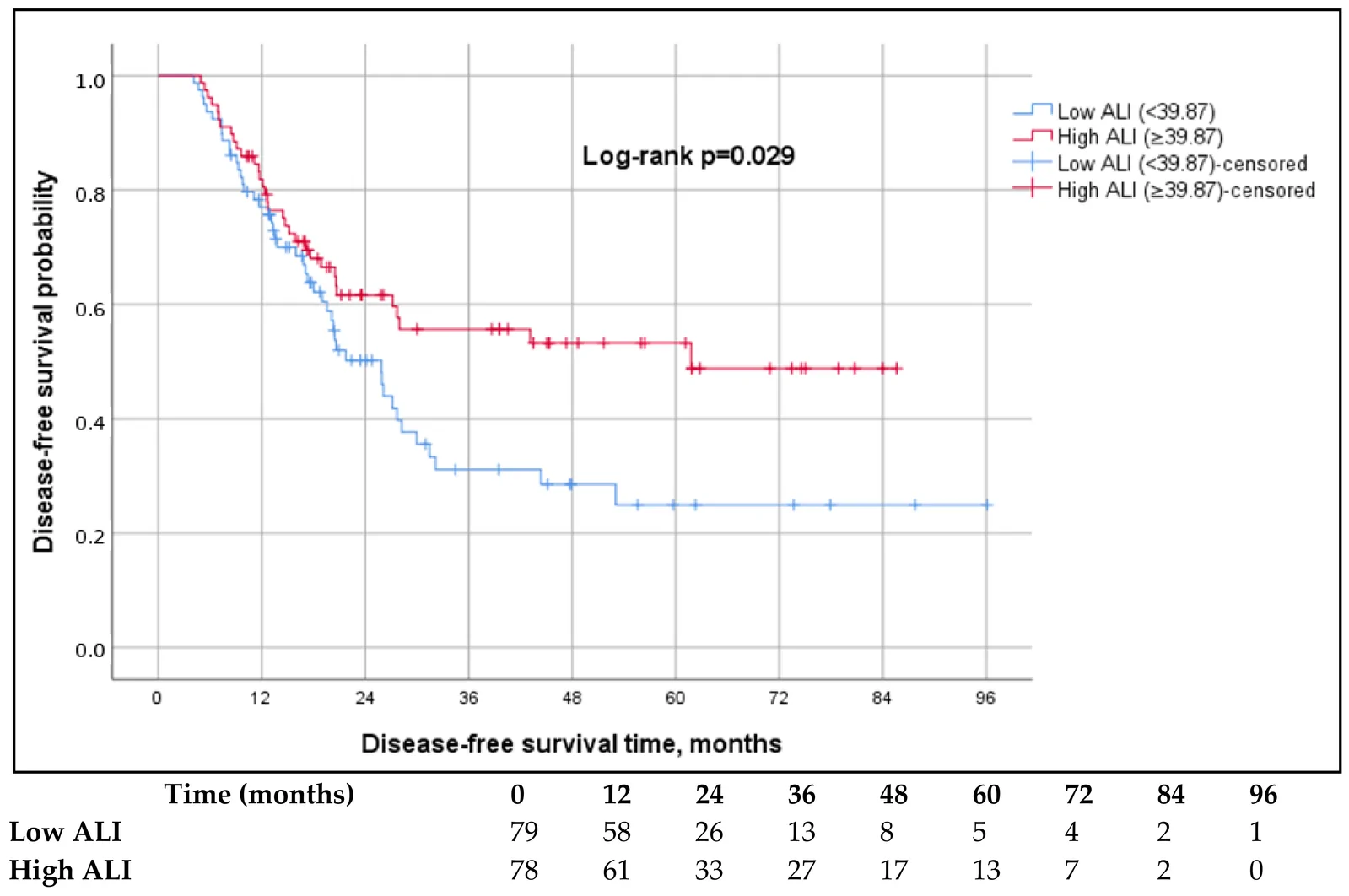
Kaplan–Meier curves for disease-free survival according to according to Advanced Lung Cancer Inflammation Index (ALI) groups.

For the Kaplan–Meier analysis, patients were stratified into low- and high-ALI groups according to the median ALI value, comprising 79 and 78 patients, respectively. Disease recurrence was observed in 46 patients (58.2%) in the low-ALI group compared with 33 patients (42.3%) in the high-ALI group. The corresponding mean DFS durations were 38.9 and 52.0 months, respectively. Kaplan–Meier curves demonstrated a significant difference in DFS between the two groups, as confirmed by the log-rank test (*p* = 0.029) (Figure 1).

Kaplan–Meier curves showed that the probability of DFS decreased earlier and more markedly in the low ALI group compared to the high ALI group. This finding supports the result from the Cox regression analysis, which found that ALI, as a continuous variable, was independently associated with DFS.

## 4. Discussion

The main finding of our study is that a low ALI score is associated with shorter disease-free survival in patients with gastric cancer receiving neoadjuvant chemotherapy, and that ALI remains an independent prognostic factor in multivariate analysis. This finding suggests that ALI, which reflects both inflammatory and nutritional status, may serve as a clinically useful biomarker for predicting the risk of recurrence in this patient group.

It is widely accepted that inflammation and malnutrition are poor prognostic factors in cancer patients. Zhang et al. evaluated 615 patients with gastric cancer who underwent gastrectomy and reported that a lower ALI score was significantly associated with more advanced TN stage, greater tumor size, and poorer DFS and OS outcomes. The study found that a high ALI score was protective for survival [12]. In another study by Pang and colleagues using propensity score matching (PSM) analysis, it was also demonstrated that a low ALI score was significantly associated with poor survival outcomes in patients with curatively resected gastric cancer. It was demonstrated that the ALI score has a higher predictive power (AUC) in predicting 3-year survival compared to other traditional inflammatory markers such as NLR, PLR (platelet-to-lymphocyte ratio), and LMR (lymphocyte-to-monocyte ratio). The meta-analysis reported in the same study, which included data from 2542 patients, further demonstrated a significant association between lower ALI values and unfavorable survival outcomes in gastric cancer [13]. Similarly, in our study, it was found that for every 10-unit increase in the ALI score among patients diagnosed with gastric cancer, there was an independent reduction in the risk of recurrence of approximately 13.3%, confirming the decisive role of inflammation and nutritional status in tumor recurrence. While studies in the literature have mostly been conducted in patient groups undergoing primary surgery or receiving postoperative treatment, our study highlights the importance of ALI in the neoadjuvant patient group and fills a significant gap.

Neoadjuvant chemotherapy can increase inflammation as a systemic stressor, thereby leading to lymphopenia, decreased albumin levels, weight loss, and ultimately cachexia. Because the ALI formula (body mass index, albumin, and lymphocytes) is directly affected, unlike the upfront surgery group—which reflects only tumor-related inflammation—it also comes to reflect the patient’s physiological tolerance to systemic treatment. Given this cumulative effect, these findings raise the possibility that ALI may have greater prognostic relevance in patients receiving neoadjuvant therapy, although direct comparative studies are required. Albumin not only reflects nutritional status but is also a negative acute-phase reactant. In cancer-related inflammation, the increase in proinflammatory cytokines suppresses hepatic albumin synthesis and increases protein breakdown, leading to the development of hypoalbuminemia. Hypoalbuminemia has been associated with malnutrition, impaired immune response, reduced tolerance to chemotherapy, and an increased risk of postoperative complications, and is considered one of the key predictors of poor prognosis. Similarly, BMI is regarded as an indirect indicator of cancer cachexia and sarcopenia. In gastric cancer, inadequate nutrient intake and increased catabolism due to inflammation lead to skeletal muscle loss and weight loss. Studies have shown that low albumin levels and a low BMI are associated with a poor prognosis in gastric cancer [15,16]. The prognostic relevance of ALI may be attributed to its capacity to capture both systemic inflammatory activity and nutritional status. By integrating these interrelated factors into a single composite measure, ALI may offer a more comprehensive assessment of a patient’s overall biological condition and prognosis. In our cohort, the association between a lower ALI score and shorter DFS is consistent with this proposed biological rationale.

Studies have shown that advanced age is associated with a poor prognosis due to factors such as an increased burden of comorbidities, reduced physiological reserve, immunosenescence, and impaired tolerance to chemotherapy. However, some researchers argue that the patient’s performance status and physiological reserve—rather than chronological age—play a more dominant role in survival by determining tolerance to chemotherapy [5]. In our study, multivariate analysis identified advanced age as an independent adverse prognostic factor for DFS, which is consistent with the literature (HR 1.027, *p* = 0.040). This finding may be explained by the cellular and immunological decline associated with aging, along with the high risk of toxicity associated with systemic chemotherapy in elderly patients.

The finding in our study that advanced clinical stage N is an independent prognostic factor for disease-free survival is consistent with the current literature on gastric cancer. Lymph node involvement is one of the most important indicators reflecting tumor burden and potential micrometastatic disease, and it ranks among the key parameters determining prognosis. Studies by Spolverato et al. and Verlato et al. have demonstrated that an advanced nodal stage has an independent adverse prognostic effect on recurrence and survival [4,5]. Chen et al. identified pathological lymph node status as one of the most robust independent predictors of prognosis among gastric cancer patients treated with neoadjuvant therapy [17]. Consistent with these findings, our study found that the risk of recurrence was approximately three times higher in patients with an advanced clinical N stage at baseline. While studies in the literature have focused on evaluating the post-treatment pathological lymph node status, our findings indicate that the pre-treatment clinical lymph node burden similarly predicts the biological behavior of the disease and the long-term risk of recurrence.

In the multivariate analysis of our study, the risk of recurrence in patients who received neoadjuvant chemotherapy other than FLOT was found to be 2.1 times higher than in the FLOT group (HR 2.116, *p* = 0.003). This finding is consistent with the Phase III FLOT4-AIO trial, which established the standard of care for neoadjuvant treatment of gastric cancer. In that study, perioperative FLOT was associated with higher rates of pathological response and R0 resection, together with a significant improvement in survival compared with ECF/ECX. These findings supported the adoption of FLOT as a standard perioperative treatment approach for locally advanced, resectable gastric and gastroesophageal junction cancers [3]. The finding in our study that non-FLOT treatment regimens are independently associated with a higher risk of recurrence supports the validity of these results from controlled clinical trials in real-world practice.

In the literature, it has been shown that in patients diagnosed with gastric cancer, clinical T stage, histological tumor grade, and serum CEA and CA19-9 levels are associated with recurrence and survival [18,19]. The association between cT stage and high tumor grade with poor disease-free survival (DFS) has been clearly emphasized in the literature [20]. Consistent with these findings, several studies have reported that elevated preoperative serum CEA and CA19-9 concentrations are associated with greater tumor burden and the presence of occult micrometastatic disease [21]. However, in a cohort study conducted specifically in gastric cancer patients who received neoadjuvant chemotherapy, it was reported that although cT stage at diagnosis, tumor grade, and pretreatment serum tumor markers were found to be significant in univariate analyses, they lost their independent prognostic value in multivariate regression models [22]. In our study, although clinical T stage, tumor grade, and CEA and CA19-9 levels were found to be associated with disease-free survival in univariate analysis, they lost their independent significance in multivariate analysis. The most likely reason for this is that the prognostic effects of these variables largely reflect tumor burden and biological aggressiveness, and therefore overlap significantly with stronger prognostic markers such as clinical N stage and ALI. Since variables are evaluated independently of one another in multivariate analysis, it is believed that the additional prognostic information provided by cT stage, tumor grade, and serum tumor markers is limited after adjusting for clinical N stage and ALI. Similarly, although the serum CEA and CA19-9 serological parameters measured at presentation initially reflect tumor burden, the effective neoadjuvant chemotherapy received by the majority of our patients—along with the cytotoxic response and downstaging it provided—may have neutralized the direct adverse effect of these markers on recurrence. Furthermore, the fact that our study included only a homogeneous group of patients with locally advanced disease who received neoadjuvant chemotherapy may have contributed to the less pronounced independent effects of these variables compared to previous studies.

The main limitations of our study include its single-center, retrospective design; the risk of selection bias due to the limited sample size of 157 patients; and the fact that dynamic changes in ALI occurring during the neoadjuvant treatment process were not evaluated. Another consideration is that post-treatment pathological variables were not incorporated into the primary prognostic model because the study was specifically designed to evaluate the prognostic value of pretreatment ALI and its potential utility as a risk-stratification marker before the initiation of neoadjuvant treatment. TRG, which reflects pathological response to neoadjuvant therapy, was therefore not included in the primary model. However, in response to this methodological consideration, we performed an additional sensitivity analysis incorporating TRG into the multivariable model. Importantly, the association between pretreatment ALI and DFS remained statistically significant after adjustment for TRG (HR = 0.873, 95% CI: 0.774–0.985; *p* = 0.028), with an effect estimate similar to that observed in the primary analysis. This finding supports the robustness of the prognostic association of pretreatment ALI with DFS even after accounting for pathological treatment response. Nevertheless, other postoperative pathological variables, including ypT and ypN stage, were not incorporated into this sensitivity analysis, and the incremental prognostic value of ALI beyond a comprehensive postoperative pathological model warrants further investigation.

Although the ROC analysis in our study found that ALI had limited discriminatory power for predicting recurrence, Cox regression analyses demonstrated that it is an independent predictor of disease-free survival. The ROC analysis evaluates only whether a recurrence has occurred or not as a binary outcome and does not take into account the time at which the recurrence occurs. In contrast, Cox regression analysis is a more appropriate method for time-based outcomes such as disease-free survival, as it evaluates the rate at which the event occurs using time-dependent survival data. Nevertheless, our study has significant strengths. To the best of our knowledge, this study is one of a limited number of studies evaluating the prognostic value of ALI on disease-free survival in gastric cancer patients who underwent neoadjuvant chemotherapy, curative gastrectomy, and D2 lymph node dissection. The inclusion of only a homogeneous patient group with locally advanced disease who underwent surgery and were managed with a similar treatment approach has enhanced the validity of the results. Furthermore, the evaluation of ALI as a continuous variable in the Cox regression model has allowed for a more accurate determination of its prognostic effect compared to analyses based solely on predefined cutoff points. Finally, the fact that ALI retained its independent prognostic value even after adjusting for important clinical variables such as age, clinical TN stage, tumor grade, serum tumor markers, and neoadjuvant treatment regimen supports the methodological strength of our study.

The inclusion of only patients who ultimately underwent curative gastrectomy after neoadjuvant chemotherapy represents an additional potential source of selection bias. Eleven patients who received neoadjuvant chemotherapy did not proceed to surgery; five declined surgery despite being considered suitable candidates for resection, whereas six developed disease progression and became unresectable. Exclusion of the latter group may have preferentially selected patients with more favorable tumor biology and response to neoadjuvant treatment. Therefore, the study cohort represents patients who successfully proceeded to curative surgery rather than the entire population initiating neoadjuvant chemotherapy, and the observed survival outcomes and prognostic effect of ALI may not be fully generalizable to all patients receiving neoadjuvant treatment.

## 5. Conclusions

Our study demonstrated that the Advanced Lung Cancer Inflammation Index (ALI) is an independent predictor of disease-free survival in patients with locally advanced gastric cancer who underwent curative gastrectomy following neoadjuvant chemotherapy. While a low ALI score was found to be associated with a higher risk of recurrence, advanced age, high clinical N stage, and a neoadjuvant treatment regimen other than FLOT were also identified as independent adverse prognostic factors. Calculated from body mass index, serum albumin level, and the neutrophil-to-lymphocyte ratio—all of which are readily available in routine clinical practice—the ALI may serve as a practical risk stratification tool that requires no additional cost, thereby contributing to the identification of high-risk patients and the individualization of postoperative follow-up strategies. However, larger, multicenter, and prospective studies are needed to validate these findings and clarify the role of ALI in clinical decision-making processes.

## Figures and Tables

**Table 1:** Baseline clinicopathological and treatment characteristics of patients receiving neoadjuvant chemotherapy for gastric cancer.

Variable	Overall Cohort, *n* = 157
Age, years, median (range)	63.55 (32.65–86.72)
Sex, *n* (%)	
Male	96 (61.1)
Female	61 (38.9)
ECOG performance status, *n* (%)	
0	82 (52.2)
1	70 (44.6)
2	5 (3.2)
Histological subtype, *n* (%)	
Non-signet ring cell carcinoma	128 (81.5)
Signet ring cell carcinoma	29 (18.5)
Grade, *n* (%)	
Grade 1	8 (5.1)
Grade 2	96 (61.1)
Grade 3	53 (33.8)
Ki-67, %, median (range)	50.0 (10–90)
HER2 status, *n* (%)	
Negative	114 (89.1)
Positive	14 (10.9)
Tumor location, *n* (%)	
Gastroesophageal junction	26 (16.6)
Fundus	50 (31.8)
Corpus/body	53 (33.8)
Antrum	28 (17.8)
Clinical T stage, *n* (%)	
cT1	8 (5.1)
cT2	52 (33.1)
cT3	67 (42.7)
cT4	30 (19.1)
Clinical N stage, *n* (%)	
cN0	21 (13.4)
cN1	64 (40.8)
cN2	39 (24.8)
cN3	33 (21.0)
Neoadjuvant chemotherapy regimen, *n* (%)	
FLOT	125 (79.6)
Non-FLOT regimen	32 (20.4)
Number of neoadjuvant cycles, median (range)	4.0 (2–8)
Tumor regression grade, *n* (%)	
TRG1	37 (23.6)
TRG2	61 (38.9)
TRG3	59 (37.6)
Number of retrieved lymph nodes, median (range)	28.0 (15–58)
CEA, median (range)	3.52 (1–580)
CA19-9, median (range)	12.0 (0–751)
Hemoglobin, g/dL, median (range)	13.3 (7.4–17.3)
White blood cell count, /µL, median (range)	7670 (4250–19,900)
Neutrophil count, /µL, median (range)	4560 (1160–17,300)
Monocyte count, /µL, median (range)	600 (100–1900)
Lymphocyte count, /µL, median (range)	1950 (640–5130)
Platelet count, /µL, median (range)	284,000 (123,000–701,000)
Albumin, g/dL, median (range)	3.95 (1.95–5.00)
BMI, kg/m^2^, median (range)	24.77 (17.51–39.11)
ALI score, median (range)	39.87 (4.79–132.58)
DFS, months, median (range)	19.53 (4.10–96.10)
Recurrence status, *n* (%)	
No recurrence	78 (49.7)
Recurrence	79 (50.3)
Final status, *n* (%)	
Alive	93 (59.2)
Deceased	64 (40.8)

Values are presented as median range or number percentage. Ki-67 data were available for 111 patients and HER2 status for 128 patients. ALI: Advanced Lung Cancer Inflammation Index; BMI: body mass index; CEA: carcinoembryonic antigen; CA19-9: carbohydrate antigen 19-9; DFS: disease-free survival; ECOG: Eastern Cooperative Oncology Group; TRG: tumor regression grade; HER2: human epidermal growth factor receptor 2; FLOT: fluorouracil, leucovorin, oxaliplatin, and docetaxel.

**Table 2:** Univariate and multivariate Cox regression analyses for disease-free survival.

Variable	Univariate HR	95% CI	*p* Value	Multivariate HR	95% CI	*p* Value
Sex, male vs. female	1.500	0.937–2.400	0.091	1.062	0.819–1.378	0.649
Age, years	1.028	1.004–1.052	0.023	1.027	1.001–1.054	0.040
ECOG performance status	0.819	0.535–1.242	0.147	—	—	—
CEA	1.005	1.003–1.007	<0.001	1.001	0.999–1.004	0.329
CA19-9	1.002	1.001–1.003	0.001	1.001	1.000–1.003	0.156
Neutrophil count	1.000	1.000–1.000	0.188	—	—	—
Lymphocyte count	1.000	0.999–1.000	0.132	—	—	—
Platelet count	1.000	1.000–1.000	0.383	—	—	—
Histological subtype, signet ring cell vs. non-signet ring cell	1.453	0.846–2.494	0.175	—	—	—
High grade, grade 3 vs. grade 1–2	2.341	1.503–3.647	<0.001	1.462	0.896–2.386	0.128
Advanced clinical T stage, cT3–4 vs. cT1–2	3.408	1.941–5.986	<0.001	1.702	0.898–3.226	0.103
Advanced clinical N stage, cN2–3 vs. cN0–1	3.601	2.245–5.777	<0.001	2.709	1.584–4.634	<0.001
ALI, per 10-unit increase	0.879	0.796–0.971	0.011	0.867	0.768–0.978	0.020
Tumor location	—	—	0.534	—	—	—
Non-FLOT neoadjuvant treatment vs. FLOT	2.639	1.644–4.238	<0.001	2.116	1.293–3.464	0.003

HR: hazard ratio; CI: confidence interval; DFS: disease-free survival; ECOG: Eastern Cooperative Oncology Group; CEA: carcinoembryonic antigen; CA19-9: carbohydrate antigen 19-9; ALI: Advanced Lung Cancer Inflammation Index; FLOT: fluorouracil, leucovorin, oxaliplatin, and docetaxel. Variables with *p* < 0.10 in univariate analysis were included in the multivariate Cox regression model. ALI was analyzed as a continuous variable per 10-unit increase.

## Data Availability

The data presented in this study are available on request from the corresponding author.

## Supplementary Materials

The following supporting information can be downloaded at: https://www.mdpi.com/article/10.3390/jcm15176806/s1, Table S1. Sensitivity analysis of factors associated with disease-free survival after additional adjustment for tumor regression grade.

## Abbreviations

ALI	Advanced Lung Cancer Inflammation Index
AUC	Area under the curve
BMI	Body mass index
CA19-9	Carbohydrate Antigen 19-9
CEA	Carcinoembryonic Antigen
CI	Confidence Interval
DFS	Disease-free survival
ECOG	Eastern Cooperative Oncology Group
HR	Hazard Ratio
LMR	Lymphocyte-to-monocyte ratio
NLR	Neutrophil-to-lymphocyte ratio
NSCLC	Non-small-cell lung cancer
PLR	Platelet-to-lymphocyte ratio
PSM	Propensity score matching
ROC	Receiver operating characteristic
TNM	Tumor size-lymph node-metastasis
TRG	Tumor regression grade
