# Supplementary material for: The Prognostic Value of the Advanced Lung Cancer Inflammation Index on Disease-Free Survival in Patients with Gastric Cancer Receiving Neoadjuvant Chemotherapy
Source: J Clin Med. 2026 Sep 2;15(17):6806. doi: 10.3390/jcm15176806 (PMC13566221; doi:10.3390/jcm15176806)
Supplement: Supplementary file 1 [file jcm-15-06806-s001.zip › jcm-4511331-supplementary.pdf]

**Table S1. Sensitivity analysis of factors associated with disease-free survival after additional adjustment for tumor regression grade.**

| **Variable** | **HR** | **95% CI** | **p value** |
|---|---|---|---|
| Sex, male vs. female | 1.082 | 0.639–1.831 | 0.770 |
| Age, years | 1.024 | 0.998–1.051 | 0.070 |
| CEA | 1.002 | 0.999–1.005 | 0.292 |
| CA19-9 | 1.001 | 0.999–1.003 | 0.234 |
| High grade, grade 3 vs. grade 1–2 | 1.362 | 0.832–2.230 | 0.220 |
| Advanced clinical T stage, cT3–4 vs. cT1–2 | 1.402 | 0.695–2.830 | 0.345 |
| Advanced clinical N stage, cN2–3 vs. cN0–1 | 2.542 | 1.451–4.454 | 0.001 |
| ALI, per 10-unit increase | 0.873 | 0.774–0.985 | 0.028 |
| Non-FLOT neoadjuvant treatment vs. FLOT | 2.101 | 1.283–3.442 | 0.003 |
| TRG2 vs. TRG1 | 2.088 | 0.875–4.984 | 0.097 |
| TRG3 vs. TRG1 | 2.165 | 0.840–5.580 | 0.110 |

HR: hazard ratio; CI: confidence interval; ALI: Advanced Lung Cancer Inflammation Index; CEA: carcinoembryonic antigen; CA19-9: carbohydrate antigen 19-9; FLOT: fluorouracil, leucovorin, oxaliplatin, and docetaxel; TRG: tumor regression grade. TRG1 was used as the reference category.
